# Long-term metabolic fate and mortality in obesity without metabolic syndrome

**DOI:** 10.1080/07853890.2022.2075915

**Published:** 2022-05-20

**Authors:** Aki Juhani Käräjämäki, Arto Korkiakoski, Janne Hukkanen, Y. Antero Kesäniemi, Olavi Ukkola

**Affiliations:** a Department of Internal Medicine, Vaasa Central Hospital, Vaasa, Finland; b Research Unit of Internal Medicine, Medical Research Center Oulu, Oulu University Hospital, University of Oulu, Oulu, Finland; c Department of Internal Medicine, Central Ostrobothnia Central Hospital, Kokkola, Finland

**Keywords:** Metabolic syndrome, obesity, metabolically healthy obesity, mortality, cardiovascular disease, atrial fibrillation

## Abstract

**Background:**

Obesity and metabolic syndrome (MetS) are known to expose to atrial fibrillation (AF), cardiovascular diseases (CVD) and mortality. Metabolically healthy obesity refers to obesity without MetS. This study aimed to investigate how obesity and MetS modify the risk of CVD, AF and mortality in very long-time follow-up.

**Methods:**

Finnish middle-aged subjects (*n* = 1045) were grouped into four subgroups according to the presence of obesity and MetS. CVD events and AF were followed for 24 years and total mortality for 30 years. Moreover, 600 available patients had a follow-up visit for metabolic examinations after approximately 22 years.

**Results:**

One-hundred and sixty-two (30%) subjects without obesity or MetS died during the follow-up. Ninety-two (17%) of the patients in this group had a CVD event and 58 (11%) were diagnosed with AF. As compared to them, obese subjects without MetS had similar metabolic fate and mortality (mortality 26 (38%), *p* = .143; CVD event 12 (18%), *p* = .858 and AF 7 (10%), *p* = .912, respectively), whereas subjects with obesity and MetS had greater mortality (102 (49%), *p* < .001), more CVD (71 (34%), *p* < .001) and AF (49 (23%), *p* < .001). Non-obese individuals with MetS had greater rates of mortality (96 (44%), *p* < .001) and CVD (80 (37%), *p* < .001), but not of AF (26 (12%), *p* = .606). Of the 40 subjects with obesity but without MetS at baseline and available for the follow-up visit, 15 (38%) were metabolically healthy at the follow-up visit.

**Conclusions:**

In the present long-term follow-up study, the presence of MetS, but not obesity only, implies a greater risk of mortality and CVD. The risk of AF is increased only in subjects with both obesity and MetS. However, obesity without MetS tends to progress eventually to obesity with MetS.

**Key messages**
1. The presence of metabolic syndrome (MetS), but not obesity only, entails a greater risk of mortality and cardiovascular diseases.
2. The risk of atrial fibrillation is increased only in subjects with both obesity and MetS.
3. Obesity without MetS tends to progress eventually to obesity with MetS.

## Introduction

Obesity and metabolic syndrome (MetS), a cluster of metabolic abnormalities [1], have high global prevalence [2,3]. These conditions are associated with atrial fibrillation (AF) [4–6], cardiovascular diseases (CVD) [7,8] and total mortality [7,9,10]. Obesity and MetS often overlap; in the literature, this is usually referred to as metabolically unhealthy obesity (MUO). However, about 35–50% of obese individuals do not have MetS [11,12]. These subjects are generally called metabolically healthy obese (MHO), although there are no universal criteria for MHO [12]. Indeed, when using the absence of MetS as a definition for MHO, subjects with ≤2 out of 5 MetS components (high systolic or diastolic blood pressure, high plasma triglycerides, low high-density lipoprotein (HDL) cholesterol, high fasting blood glucose and abdominal obesity) can be classified as MHO. Thus, MHO does not necessarily imply entirely metabolically healthy subjects, but persons with fewer cardiometabolic risk factors than those with MUO [12].

The mechanisms that separate MHO and MUO are not fully understood, but, for instance, genetics, gut microbiome and physical activity may play central roles, as discussed in detail elsewhere [12–14]. MHO individuals tend to be younger, more often female, smoke less and exercise more than their MUO counterparts [15]. Whether MHO and MUO are two distinct subgroups or whether there is a continuum from simple obesity without any cardiometabolic abnormalities to a growing number of MetS components and, finally, MUO, is under debate [12]. Apparently, however, about half of the MHO patients convert to MUO within 1–2 decades [16–20], whereas opposite transition from MUO to MHO occurs in only about 10–20% of the MUO patients within 20 years [16,17]. As a result, MHO is often seen as a precursor of MUO and has even been called a “honeymoon phase” of obesity [13].

There are only a few reports with diverging results about the risk of AF in MHO patients in comparison with MUO patients [21–24], whereas, according to a recently published meta-analysis of 23 prospective studies with almost 4.5 million patients, the risk of CVD and overall mortality are both about 1.6-fold higher in MHO subjects as compared to metabolically healthy subjects with normal body mass index (BMI 18.5–24.9 kg/m^2^). In the same study, though, metabolic abnormality conferred even greater, about 3-fold, risk of CVD than obesity alone. No clear dose–response was observed between the number of MetS components and the risk of CVD, possibly explained by the small number of studies with detailed MetS data [25].

Because the lack of universal criteria to MHO and to emphasize that not all subjects with obesity but without MetS are really metabolically healthy, we do not call obesity without MetS as MHO in the present article. Instead, the study cohort was grouped to patients with neither MetS nor obesity (M-O-), with only MetS (M+O-), with only obesity (M-O+) and persons with both conditions (M+O+). We aimed to investigate in Finnish middle-aged population whether these groups entail a different risk of total mortality, CVD and AF as compared to the reference group. We also investigate the long-term stability of the metabolic status in these patients. To the best of our knowledge, there are no prior studies providing follow-up data over such a long period and with equally detailed baseline examinations as in this study.

## Material and methods

This study is based on the OPERA cohort originally launched in the early 1990s to study the risk factors of atherosclerotic CVD [26], with 300 men and 300 women, aged 40–60 years, with hypertension diagnosis and a verified need for antihypertensive medication, and 1:1 age- and sex-matched controls without hypertension diagnosis. All study participants were randomly selected from the National Health Register among the inhabitants of the city of Oulu in Northern Finland. Of a total of 1200 individuals, 1045 (87%) accepted the invitation and participated in the study. There were 520 men (261 from the hypertension group and 259 from the control group) and 525 women (258 from the hypertension group and 267 from the control group).

All study participants underwent comprehensive baseline investigations in the research laboratory of the Department of Internal Medicine, University of Oulu, with blood tests, height and weight measurements, standardized health questionnaires and ECG registration, performed by two specially trained nurses. Moreover, ultrasonographical liver–kidney contrast evaluation [27] was performed to assess hepatosteatosis by a trained radiologist with more than 10 years’ experience of abdominal ultrasound examinations. Hewlett-Packard 77020 A ultrasound colour system for M-mode, two-dimensional and Doppler examinations were used to perform the echocardiographic measurements by one experienced cardiologist. The left ventricular mass was assessed [28] and the left ventricular mass index was calculated by dividing left ventricular mass by body surface area. The radiologist and cardiologist were blinded to the other data. All these examinations were performed after an overnight fast and took place during the years 1990–1993.

MetS was diagnosed in a subject with any three out of five different cardiovascular risk factors (waist >94 cm in men or >80 cm in women; plasma triglycerides >1.7 mmol/L; HDL cholesterol  < 1.03 mmol/L in men or  < 1.30 mmol/L in women; blood pressure >130/85 mmHg or treatment for hypertension; fasting plasma glucose > 5.6 mmol/L or treatment for hyperglycaemia) (1) The subjects were diagnosed to have impaired fasting glucose with fasting glucose 6.1–6.9 mmol/L, impaired glucose tolerance with 2-h glucose in the oral glucose tolerance test 7.8–11.0 mmol/L, diabetes if the patient had a previous diabetes diagnosis or fasting glucose was ≥ 7.0 mmol/L or 2-h glucose in the oral glucose tolerance test >11 mmol/L or both. If the patient did not have any of these, the patient was considered normoglycaemic. An individual with BMI ≥30 kg/m^2^ was considered to have obesity.

Mortality data were based on the National Death Registry and the diagnoses of cardiovascular events on the hospital discharge registry of the National Institute for Health and Welfare. Cardiovascular events included a coronary heart disease event and stroke, whichever occurred first. The coronary heart disease diagnosis was made on the following diagnoses: I20.0, I21, I22 [International Statistical Classification of Diseases and Related Health Problems (ICD-10)]/410, 4110 [ICD-8/9] as the main diagnosis (symptom or cause) and I21, I22 [ICD-10]/410 [ICD-8/9] as a first or second side diagnosis (symptom or cause) and third side diagnosis (ICD-8/9 only) or if the subject had undergone coronary artery bypass grafting or coronary angioplasty. CHD as a cause of death included I20–I25, I46, R96, R98 [ICD-10]/410–414, 798 (not 7980 A) [ICD-8/9] as the underlying cause of death or immediate cause of death and I21 or I22 [ICD-10]/410 [ICD-8/9] as first to third contributing cause of death. Stroke included I61, I63 (not I63.6), I64 [ICD-10]/431, 4330 A, 4331 A, 4339 A, 4340 A, 4341 A, 4349 A, 436 [ICD-9]/431 (except 43101, 43191) 433, 434, 436 [ICD-8] as main diagnosis (symptom or cause) or as a first or second side diagnosis (symptom or cause) or as a third side diagnosis (ICD-8/9 only) or as an underlying cause of death or immediate cause of death or as a first to third contributing cause of death. The diagnosis of AF (including atrial flutter) was based on standard 12-lead resting ECG and made if this event was listed as ICD-10 code (I48) in the hospital discharge registry and/or in the National Death Registry (follow-up). At baseline, there was one patient with AF diagnosis. This patient had both MetS and obesity.

The total mortality was assessed until 31 December 2020, that is, up to 30 years (mean 25 years) and CVD and AF until 31 December 2014, which equals up to 24 years. As the first CVD and AF events accrued differentially, the mean follow-up was 19.5 years for CVD and 20 years for AF (mean 19.5 years with CVD and 20 years with AF).

Study participants (*n* = 600) visited the research laboratory of the Department of Internal Medicine, University of Oulu, during the years 2013–2014, about 22 years after the study enrolment. Among control examinations, a 2-h oral glucose tolerance test and height and weight measurements were performed. The metabolic and obesity status were redefined according to these measurements and the data obtained from the medical records.

All study subjects gave written informed consent for the use of their medical records. The Ethics Committee of the Northern Ostrobothnia Hospital District (Oulu, Finland) approved the study (48/2009).

### Statistics

Chi-square test was used to compare the statistical significance of differences in categorical variables and ANOVA test (parametric distribution) or Kruskall–Wallis (non-parametric distribution) test in continuous variables. The normality of data distribution was assessed by Shapiro–Wilk test (data with Shapiro–Wilk test > 0.05 was assessed to be in normal distribution). Cox hazards model and Cox multivariate regression model with clinically relevant, non-MetS related variables were used to investigate the predictive value of the study groups on the follow-up events. The cumulative proportional probabilities for survival were expressed by using the Kaplan–Meier analysis. The log-rank test was performed to examine the statistical significance of the separation of the curves. A *p* value  < 0.05 was considered statistically significant. Statistical analyses were performed by IBM SPSS Statistics for Windows version 27 (IBM, Armonk, NY).

## Results

In this study, there were 548 (52%) individuals in the M-O- group, 219 (21%) in the M + O- group, 68 (7%) in the M-O + group and 210 (20%) in the M+O + group. Thus, of all obese patients, 24% were in the M-O + group. Of these subjects, 63 (23%) had two MetS components and 5 (2%) one MetS component. None of the M-O + patients was without any MetS component. The baseline characteristics of the study participants are presented in Table 1.

**Table 1.** The baseline characteristics of the study participants by study group.

	M-O- (*n* = 548)	M + O- (*n* = 219)	M-O+ (*n* = 68)	M + O+ (*n* = 210)	*p* Value	*p* Value <.05
**Patient characteristics**						
Sex (female), *n* (%) (*n* = 1045)	309 (56%)	88 (40%)	33 (49%)	95 (45%)	<.001	A, C
Age (years) (*n* = 1045)	51 ± 6	51 ± 6	52 ± 6	52 ± 6	.187	
Smoking (pack years) (*n* = 1045)	8 ± 12	11 ± 15	12 ± 14	13 ± 17	<.001	A, B, C
Alcohol consumption (g/week) (*n* = 1045)	51 ± 73	75 ± 108	60 ± 95	73 ± 105	.289	
**Metabolic data**						
Diagnosis of hypertension after baseline examinations, *n* (%) (*n* = 1045)	205 (37%)	134 (61%)	39 (57%)	162 (77%)	<.001	A, B, C, E, F
Coronary artery disease, *n* (%) *n* = 1045	31 (6%)	29 (13%)	4 (6%)	22 (10%)	<.001	A, C
Fatty liver, *n* (%) (*n* = 1028)	45 (8%)	83 (39%)	19 (28%)	134 (65%)	<.001	A, B, C, E, F
Glycaemic control (*n* = 1041)						
Normoglycaemia, *n* (%)	479 (88%)	132 (61%)	59 (87%)	89 (43%)	<.001	A, C, D, E, F
Impaired fasting glucose, *n* (%)	2 (0%)	4 (2%)	0 (0%)	8 (4%)		
Impaired glucose tolerance, *n* (%)	51 (9%)	48 (22%)	7 (10%)	56 (27%)		
Diabetics, *n* (%)	14 (3%)	34 (16%)	2 (3%)	56 (27%)		
MetS criteria, waist, n (%) *n* = 1044	147 (27%)	179 (82%)	64 (94%)	210 (100%)	<.001	A, B, C, D, E, F
MetS criteria, triglycerides, *n* (%), *n* = 1045	30 (5%)	155 (71%)	1 (1%)	138 (66%)	<.001	A, C, D, F
MetS criteria, HDL, *n* (%), *n* = 1045	52 (9%)	140 (64%)	5 (7%)	119 (57%)	<.001	A, C, D, F
MetS criteria, fasting glucose, *n* (%), *n* = 1045	31 (6%)	78 (36%)	1 (1%)	115 (55%)	<.001	A, C, D, E, F
MetS criteria, blood pressure, *n* (%), *n* = 1045	422 (77%)	212 (97%)	60 (88%)	207 (99%)	<.001	A, B, C, D, F
MetS (by IDF criteria), *n* (%) *n* = 1045	0 (0%)	179 (82%)	0 (0%)	210 (100%)	<.001	A, C, D, E, F
Waist (cm) (*n* = 1042)	83 ± 9	93 ± 9	101 ± 12	106 ± 10	<.001	A, B, C, D, E, F
Body mass index (kg/m^2^) (*n* = 1045)	24.9 ± 2.4	27.0 ± 2.1	33.1 ± 3.2	34.0 ± 3.7	<.001	A, B, C, D, E
Blood pressure, systolic (mmHg) *n* = 1045	144 ± 22	150 ± 20	152 ± 23	158 ± 22	<.001	A, B, C, E, F
Blood pressure, diastolic (mmHg) *n* = 1045	86 ± 12	91 ± 11	92 ± 12	93 ± 11	<.001	A, B, C, E
**Echocardiographic data**						
Left ventricular mass (g) (*n* = 945)	223 ± 77	255 ± 77	286 ± 89	300 ± 93	<.001	A, B, C, D, E
Left ventricular mass index (g/m^2^) (*n* = 945)	125 ± 37	134 ± 39	140 ± 39	145 ± 39	<.001	A, B, C, E
Left atrial diameter (mm) (*n* = 896)	37 ± 5	39 ± 4	42 ± 4	43 ± 5	<.001	A, B, C, D, E
Fractional shortening (%) (*n* = 945)	35 ± 6	35 ± 6	35 ± 5	35 ± 6	.929	
**Laboratory parameters**						
ALT (U/L) *n* = 1042	25 ± 12	38 ± 27	35 ± 20	44 ± 32	<.001	A, B, C, E, F
GGT (U/L) *n* = 1044	31 ± 26	66 ± 116	45 ± 39	69 ± 85	<.001	A, B, C, D, E, F
Fasting glucose (mmol/L) *n* = 1045	4.3 ± 0.6	5.0 ± 1.7	4.3 ± 0.5	5.7 ± 2.3	<.001	A, C, D, E, F
Fasting insulin (mU/L) *n* = 1045	9.2 ± 5.8	15.2 ± 9.4	15.0 ± 9.2	23.2 ± 16.0	<.001	A, B, C, E, F
HOMA-IR *n* = 1045	1.8 ± 1.3	3.5 ± 2.9	2.9 ± 1.9	6.0 ± 5.3	<.001	A, B, C, E, F
Cholesterol (mmol/L) *n* = 1045	5.5 ± 1.0	6.0 ± 1.1	5.6 ± 1.1	5.8 ± 1.0	<.001	A, C, D, E
LDL-cholesterol (mmol/L) *n* = 1045	3.4 ± 0.9	3.8 ± 0.9	3.5 ± 1.0	3.6 ± 0.9	<.001	A, C, D, E
HDL-cholesterol (mmol/L) *n* = 1045	1.5 ± 0.4	1.1 ± 0.3	1.4 ± 0.3	1.1 ± 0.3	<.001	A, C, D, F
Triglycerides (mmol/L) *n* = 1045	1.1 ± 0.4	2.2 ± 1.2	1.2 ± 0.3	2.3 ± 1.3	<.001	A, C, D, F
Creatinine (µmol/L) *n* = 1043	80 ± 15	89 ± 63	80 ± 14	83 ± 14	<.001	A, C, D
GFR-Epi (mL/min) *n* = 1043	85 ± 15	83 ± 16	85 ± 15	84 ± 15	.804	
Hs-CRP (mg/L) *n* = 1036	2.4 ± 4.9	4.9 ± 11.6	3.4 ± 3.4	6.4 ± 7.5	<.001	A, B, C, E, F
Adiponectin (mg/L), *n* = 1041	17.7 ± 7.2	13.8 ± 6.0	16.2 ± 6.5	13.2 ± 5.3	<.001	A, C, D, F
**Medication**						
Lipid lowering drugs, *n* (%) *n* = 1045	9 (2%)	11 (5%)	1 (1%)	9 (4%)	.035	A, C
Antihypertensive medication, *n* (%) *n* = 1045	202 (37%)	139 (63%)	37 (54%)	164 (78%)	<.001	A, B, C, E, F
Acetylsalicylic acid, *n* (%) *n* = 1045	22 (4%)	19 (9%)	3 (4%)	14 (7%)	.065	

The continuous variables are expressed as mean values ± standard deviation and the categorical variables as absolute numbers with percent in brackets. The differences were tested by the ANOVA test for the parametric continuous variables, Kruskall–Wallis for the non-parametric continuous variables, and Pearson chi-squared test for categorical variables. All laboratory values were taken after an overnight fast. The statistical difference for continuous variables between the groups was analysed with Tukey’s method. A *p* value <.05 was considered statistically significant. The pairwise comparisons with *p* value <.05: A, M-O- and M + O-; B, M-O- and M-O+; C, M-O- and M+O+; D, M+O- and M-O+; E, M+O- and M+O+; F, M-O + and M+O+.

The abbreviations are as follows: ALT: alanine aminotransferase; BMI: body mass index; GFR-Epi: glomerulus filtration rate by the Chronic Kidney Disease Epidemiology Collaboration equation; GGT: gamma-glutamyl transpeptidase; HDL: high-density lipoprotein; HOMA-IR: homeostatic model assessment of insulin resistance; hs-CRP: high sensitive C-reactive protein; IDF: International Diabetes Federation; LDL: low-density lipoprotein; MetS: metabolic syndrome; M-O-: no metabolic syndrome or obesity; M + O-: metabolic syndrome without obesity; M-O+: obesity without metabolic syndrome; M+O+: metabolic syndrome and obesity

### Total mortality

**Figure 1.**
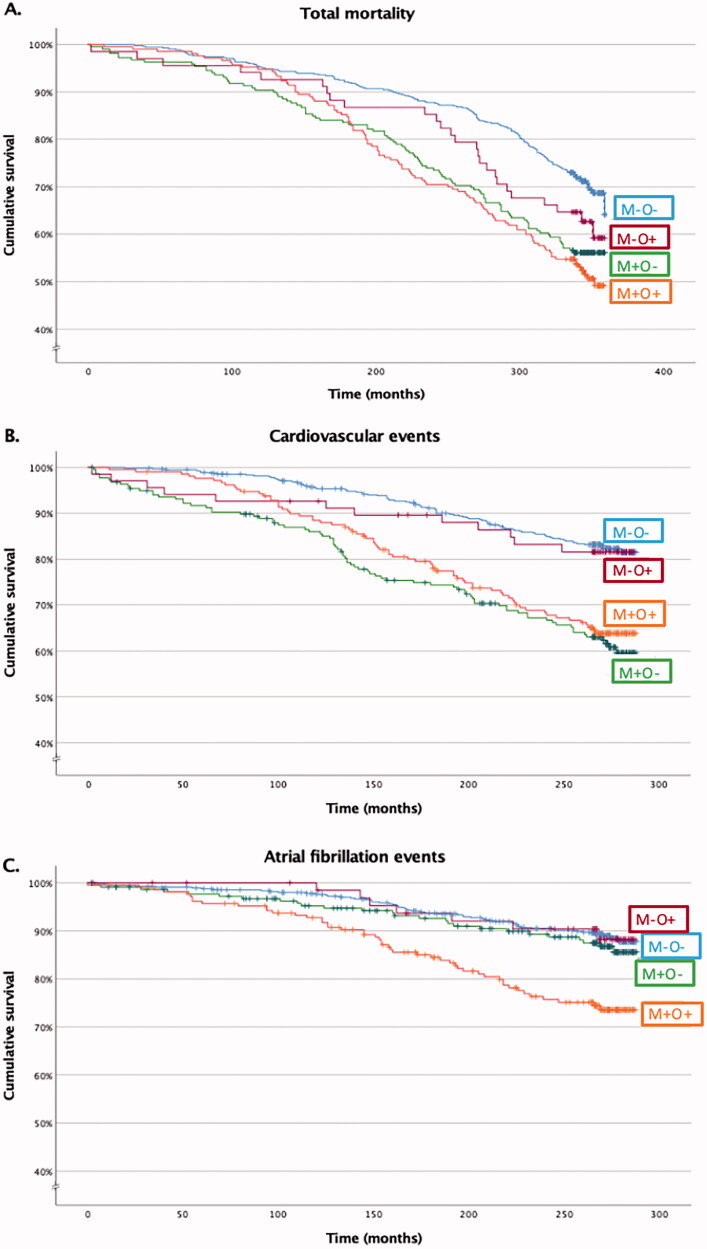
The cumulative survival of total mortality (A), cardiovascular (B) and atrial fibrillation (C) in the study groups. The curve separations were tested by log rank test and were statistically significant in 1 A between M-O- and M+O-***, M-O- and M+O+*** in 1B between M-O- and M+O-***, M-O- and M+O+***, M-O + and M+O-**, M-O + and M+O+* in 1 C between M-O- and M+O+***, M+O- and M+O+**, M-O + and M+O+*. M: metabolic syndrome; O: obesity; -: condition does not exist; +: condition exists. *log-rank < 0.05, **log-rank < 0.01, ***log-rank < 0.001.

Altogether 162 (30%) patients from the M-O- group, 96 (44%) from the M+O- group, 26 (38%) from the M-O + group and 102 (49%) from the M+O + group died during the follow-up (*p* < 0.001). The differences in total mortality rates were statistically significantly different between M-O- and M+O- (*p* < 0.001) as well as M-O- and M+O+ (*p* < 0.001), but not between M-O- and M-O+ (*p* = 0.143), M+O- and M+O+ (*p* = 0.325), M+O- and M-O+ (*p* = 0.414) or M-O + and M+O+ (*p* = 0.137). Figure 1(A) shows the survival curves in the subgroups (log-rank < 0.001). Hazard ratios (HRs) with multivariate Cox regression analyses with M-O- as reference group are shown in Table 2, whereas supplementary Table 1 depicts all pair-wised HRs. During the follow-up in the M + O- group, men had higher risk of death than women (74 (56%) *vs.* 22 (25%), *p* < .001), as well as those with fatty liver as compared to those without fatty liver (45 (54%) *vs.* 47 (36%), *p* = .007) and higher LVMI tertile (17 (25%) *vs.* 29 (43%) *vs.* 41 (60%), *p* < .001, respectively), whereas never-smokers had fewer deaths than current or former smokers (32 (35%) *vs*. 64 (50%), *p* = 0.022). No variables were found to predict death statistically significantly in the M+O+ group during the follow-up.

**Table 2.** Mortality, cardiovascular events and atrial fibrillation in the study groups.

	M-O- (*n* = 548)	M+O- (*n* = 219)	M-O+ (*n* = 68)	M+O+ (*n* = 210)
**Total mortality rate**	162 (30%)	96 (44%)	26 (38%)	102 (49%)
**Hazard ratio**	–	1.729 (1.343–2.226)***	1.390 (0.918–2.103)	1.961 (1.530–2.514)***
**Multivariable hazard ratio, model 1**	–	1.368 (1.052–1.779)*	1.010 (0.661–1.543)	1.577 (1.219–2.038)**
**Multivariable hazard ratio, model 2**	–	1.414 (1.055–1.897)*	1.079 (0.684–1.701)	1.450 (1.056–1.991)*
**Cardiovascular events**	92 (17%)	80 (37%)	12 (18%)	71 (34%)
**Hazard ratio**	–	2.637 (1.954–3.559)***	1.087 (0.596–1.985)	2.341 (1.717–3.192)***
**Multivariable hazard ratio, model 1**	–	2.089 (1.535–2.841)***	0.833 (0.453–1.529)	1.951 (1.419–2.683)***
**Multivariable hazard ratio, model 2**	–	2.127 (1.517–2.981)***	0.630 (0.315–1.262)	1.533 (1.049–2.240)*
**Atrial fibrillation incidence**	58 (11%)	26 (12%)	7 (10%)	49 (23%)
**Hazard ratio**	–	1.226 (0.772–1.947)	0.984 (0.449–2.156)	2.557 (1.747–3.743)***
**Multivariable hazard ratio, model 1**	–	1.120 (0.692–1.812)	0.779 (0.353–1.717)	2.235 (1.516–3.295)***
**Multivariable hazard ratio, model 2**	–	0.977 (0.562–1.699)	0.582 (0.253–1.339)	1.625 (0.959–2.752)

All hazard ratios are calculated with M-O- as the reference group. Multivariable hazard ratio model 1 is adjusted for age, sex, smoking (pack years), amount of alcohol consumption and LDL cholesterol. Multivariable hazard ratio 2 is adjusted for multivariable hazard ratio model 1 + hs-CRP, adiponectin and left ventricular mass index (total mortality and cardiovascular events)/left atrial diameter (atrial fibrillation).

M-O-: no metabolic syndrome or obesity; M+O-: metabolic syndrome without obesity; M-O+: obesity without metabolic syndrome; M+O+: metabolic syndrome and obesity.

*p < .05, **p < 0.01, ***p < .001.

### Cardiovascular diseases

In the MHNO group, there were 92 (17%) patients, in the M+O- group 80 (37%), in the M-O + 12 (18%) and in the M+ O+ group 71 (34%) who suffered a CVD event during the follow-up time (*p* < .001). Thus, M-O- and M-O+ patients had fewer CVD events than M+O- (*p* < .001 and *p* = .004, respectively) and M+O + patients (*p* < .001 and *p* = .011, respectively), whereas there was no statistically significant difference between M-O- and M-O+ patients (*p* = .858) or M+O- and M+O+ patients (*p* = .555). Thereby, MetS, not obesity, was associated with CVD events as presented in the survival curve in Figure 1(B) (log-rank <0.001). Table 2 presents HRs of CVD with multivariate Cox regression analyses between the groups. Within the M + O + group, men had more CVD events than women (48 (42%) *vs.* 23 (24%), *p* = .008), as well as those with fatty liver (with fatty liver 56 (42%) *vs.* without fatty liver 15 (21%), *p* = .003) and higher LVMI tertiles (10 (19%) *vs.* 19 (36%) vs. 24 (44%), *p* = .014, respectively). Likewise, within the M + O- group, the risk of CVD event was higher in men (55 (42%) *vs.* 25 (28%), *p* = .041) and in higher LVMI tertiles (16 (24%) *vs.* 24 (36%) *vs.* 34 (50%), *p* = .007), respectively).

### Atrial fibrillation

During the follow-up, there were 58 (11%) patients in the M-O- group, 26 (12%) in the M+O- group, 7 (10%) in the M-O+ group and 49 (23%) in the M+O + group diagnosed with AF (*p* < .001). With the M+O+ group, the difference was significant as compared to the M-O- group (*p* < .001), as well as the M+O- (*p* = .002) and the M-O+ patients (*p* = .020), while there were no statistically significant differences between other groups. Figure 1(C) shows the cumulative survival of AF between the study groups. HRs with multivariate Cox regression analyses between the groups are shown in Table 2. As seen in the table, after left atrial diameter (among parameters of systemic inflammation) was included the analysis, the risk of AF among M+O+ patients as compared to M-O- patients and M+O- patients, but not M-O+ patients, was alleviated to statistically insignificant.

### Follow-up visit

**Figure 2.**
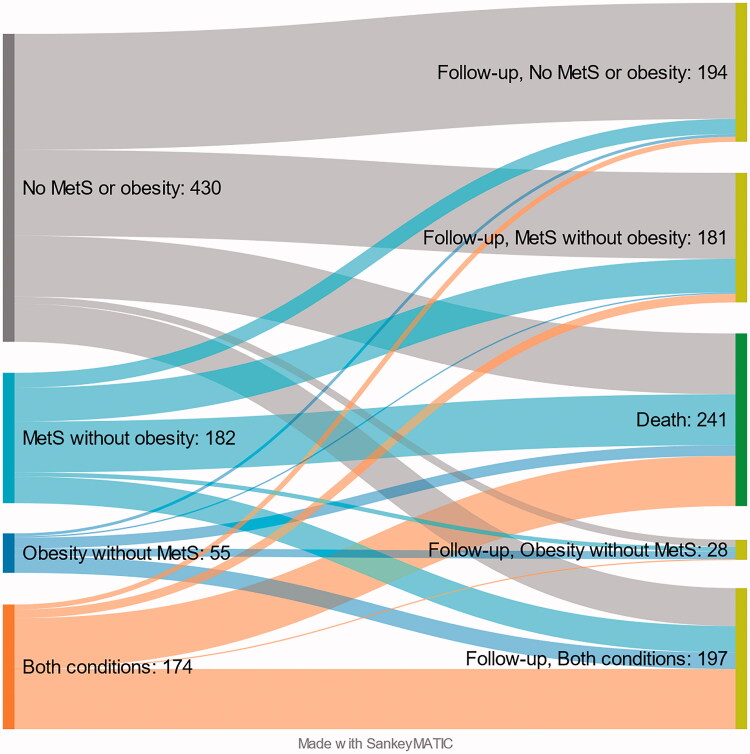
The Sankey diagram of death rates and metabolic sustainability and transition between the groups. The subjects who were still alive at the time of the control visit (31 December 2014) but had not attended the control visit were excluded from the graph. MetS, metabolic syndrome.

There were 345/548 (63%) subjects from the M-O- group, 111/219 (51%) from the M+O- group, 40/68 (59%) from the M-O+ group and 104/210 (50%) from the M+O+ group available for the control visit approximately 22 years after the enrolment. After re-grouping the 600 study participants who underwent the control visit, there were 194 (32%) patients in the M-O- group, 181 (30%) patients in the M+O- group, 28 (5%) in the M-O+ group, and 197 (33%) in the M+O+ group. At baseline, the percentages were 52%, 21%, 7% and 20%, respectively. These and the death rates in each group are illustrated in the Sankey flow diagram in Figure 2, and Supplementary Table 2 depicts in detail the persistence of group designation and transfers between groups among the subjects who attended the control visit. Overall, there was a tendency towards MetS and obesity over time. A considerable proportion of original M-O- subjects converted into M+O- subjects and original M+O- and M-O+ individuals into M+O+ individuals.

## Discussion

This study demonstrates that M-O+ subjects did not have increased risk of AF, CVDs or mortality as compared to the reference group M-O-. By contrast, subjects with MetS, whether obese or not, had increased risk of total mortality and CVDs, whereas only M+O+ patients were at greater risk of AF than the patients in the reference group. However, M-O + seems to be a relatively unstable condition with a tendency to convert to M+O+ over time.

Although our study did not show any difference in the CVD risk between M-O- and M-O+ individuals, the most recent meta-analysis, covering 21 studies (mostly prospective) with nearly 4.5 million participants, reported approximately 1.6-fold risk of future CVD events among MHO subjects as compared to metabolically healthy normal weight subjects [25]. Moreover, as compared to the healthy normal weight subjects, the authors demonstrated about 1.5-fold CVD risk among overweight/obese subjects without any metabolic abnormalities, and there was no graded increase in CVD risk by the number of MetS components (from 0 to 2). Still, within the study group consisting of normal-weight individuals, metabolic abnormalities implied even greater CVD risk (about 3.1-fold) as compared to individuals without either MetS or obesity. Notably, in this meta-analysis, subjects with obesity and overweight were not included in the same study group but were separately compared with normal weight subjects [25]. The same meta-analysis also reported that all-cause mortality was 1.6-fold and CVD mortality 2.2-fold in MHO subjects compared with metabolically healthy normal weight subjects [25]. A very recent prospective paper covering about 380,000 British patients who were followed by over 11 years reported quite similar results as total mortality and the risk of CVD were about 1.2-fold greater among MHO subjects than metabolically healthy subjects without obesity [29]. In a cohort with 3.5 million participants but with mean follow-up of only 5.4 years, the CVD risk was reported to be about 1.5-fold higher among MHO subjects as compared to normal weight individuals without metabolic abnormalities, but conflicting with the above-mentioned meta-analysis, the risk was associated with the number of metabolic abnormalities at baseline [30]. A Chinese 11-year follow-up study with more than 93,000 participants showed that MetS, but not obesity or overweight, predicts higher all-cause mortality rates in middle-aged population [31]. It is also reported that if subjects have stable MHO, the risk of CVD is not different from metabolically healthy lean subjects [18]. One explanation for the discrepant results may be found in a meta-analysis by Zheng et al. which did not find a difference in CVD events between MHO and metabolically healthy normal weight subjects in studies with follow-up duration of less than 10 years. However, when only studies with over 10 years’ follow-up were included, there was a clear, approximately 1.6-fold risk of future CVD events among MHO subjects as compared to the reference group [32]. In line with this, a 30-year follow-up study of middle-aged Swedish men reported that the risk of CVD events and overall mortality is increased by increasing BMI category, and within each category, the subjects with MetS are at greater risk than the subjects without MetS [33]. It is interesting, however, that in this study no differences between M-O+ patients and patients in the reference group were found in CVD survival even after up to 24 years of follow-up, and that in a large Korean 9-year follow-up study (with BMI ≥ 25 kg/m^2^ considered obese [34], the CVD risk among initially metabolically unhealthy patients with obesity did not change if they lost weight but remained metabolically unhealthy during the follow-up – or *vice versa*. Moreover, individuals with MHO at baseline who developed MetS during the follow-up had increased CVD risk as compared to stable MHO patients, but if initial MHO patients lost weight and became metabolically healthy non-obese subjects, the CVD risk was not statistically different from stable MHO subjects. However, converting metabolic status from unhealthy to healthy whether the subject had obesity or not, reduced CVD risk considerably [35]. Thus, one of the main conclusions from this study, namely that MetS, not body weight, is the decisive risk factor for CVD events, is in line with these earlier findings, even though improvements in the subclinical signs of atherosclerosis are reported in MHO subjects after lifestyle modification and weight loss [36,37]. Finally, MetS rather than obesity is related to myocardial dysfunction [38], which may party explain the higher CVD morbidity among the individuals with MetS than those without MetS.

There are four earlier longitudinal population-based studies about how obesity and metabolic status modify the risk of AF. Feng et al. followed nearly 48,000 Norwegian adults for about 8 years. In contrast to this study, the study concluded that the risk of AF correlates with obesity irrespective of metabolic status [22]. Meanwhile, in a Swedish 14-year follow-up study, overweight subjects with MetS as well as obese subjects with or without MetS were at increased risk of having AF as compared to normal weight subjects without MetS [21], whereas a large Korean retrospective study with 7.5 years’ follow-up and nearly 390,000 participants demonstrated that although obesity was an independent predictor of AF, individuals with MetS seemed to have the greatest risk for AF as non-obese subjects with MetS were at a greater risk of AF than obese subjects without MetS [23]. Notably, though, in that study the mean patient age was somewhat younger than in our study. Additionally, the diagnostic limit for obesity is lower in Asia (27.5 kg/m^2^) than in Europe because of the different association between BMI and health risks [34]. Thus, the results from this study are different as it reports that only M + O + individuals were at increased risk for AF as compared to the reference group, M-O-. The divergent results between the studies mentioned above and our study may be explained by differences in study cohorts, the varying diagnostic criteria of MHO and, above all, the duration of follow-up. Namely, the incidence of AF increases steeply with advancing age [39], so that if follow-up time does not come up to senior citizen age, the differences between the groups may not become evident. This may also partly explain the results of the register study by Fauchier et al., who followed nearly 2.9 million French people for about 5 years, after which they concluded that obesity was an independent risk factor for AF and the risk of AF increased by advancing number of metabolic abnormalities in subjects with obesity as well as subjects without obesity [24]. Notably, though, in this cohort the subjects with MetS were about 15–20 years older than those without MetS. Thus, although age-adjusted, the analysis may not take into full consideration how firmly advancing age increases the risk of AF. However, the reported clear link between obesity and increased risk of future AF is different to the result in this study. To the best of our knowledge, this study offers the longest follow-up about the association between AF and individuals classified by their metabolic status and the presence of obesity. Indeed, in our study, after approximately 15 years the AF survival curve of the M+O+ group began to separate distinctly from the other curves. While the other studies [22–24] are registry studies, this study and the study by Nyström et al. [21] provide detailed baseline examinations, our study being the only study presenting baseline echocardiographic data. As demonstrated in the multivariable HR models, this data suggests that increased left atrial diameter, one of the main drivers of AF [40,41], is the decisive factor behind the greatest risk of AF among M+O+ patients, especially when M+O+ patients are compared to M-O- patients or M+O- patients. However, more long-term studies about the association between AF and metabolic status among subjects with obesity are needed.

In MHO studies the following factors should be noticed. First, whether obesity without MetS is a distinct condition or just a part of a continuum towards obesity with MetS is still somewhat undetermined. It has long been known, however, that increasing age is a risk factor for MetS [42], which is emphasized among individuals with obesity as about half of subjects with MHO convert to MUO within one to two decades [16–20]. Indeed, our study is in line with these reports as nearly two-thirds of the original M-O+ patients were diagnosed to have MetS after about two decades of follow-up. Thus, as shown earlier, long-term stability of metabolic health among MHO subjects is the exception, not the rule [17,43,44]. Moreover, the longer the obesity duration or the greater the visceral abdominal fat accumulation, the greater the risk of MHO converting into MUO [43,45]. In fact, our findings agree with this position, because although there was no difference in BMI between M-O+ and M+O+ groups, waist circumference among M-O+ subjects was significantly lower as compared with M+O+ subjects. Indeed, for a great proportion of MHO patients the metabolically healthy phenotype is only a transient condition that converts later into metabolically unhealthy phenotype. Therefore, the duration of follow-up may have a great impact on the results of MHO studies. Another factor that impacts the results is the criteria used to define MHO. For example, if MHO is considered as obesity without MetS, but including ≤ 2 components of MetS, hepatosteatosis, another CVD risk factor, or even existing CVD, the morbidity and mortality risks are likely to be quite different than if MHO is defined as obesity without any additional cardiometabolic risk factors. Thus, by not calling individuals with obesity but not MetS as MHO individuals, as often referred in the prior literature, but rather M-O + individuals, we wish to emphasize that not all of them were totally metabolically healthy. Additionally, in some MHO studies overweight subjects without obesity or MetS may be included in the MHO group, whereas in some papers these subjects are classified to the reference group. As mentioned earlier, the BMI limit for obesity recommended by WHO in Asian populations is also lower than elsewhere [34]. Finally, although BMI is used to define obesity, it does not equal excess visceral adiposity, metabolically the most harmful obesity phenotype [46].

Although it still is somewhat controversial as to what degree of CVD risk MHO exposes to, MHO is not a benign condition for all MHO subjects. It is likely that there is a subpopulation among MHO subjects at increased CVD risk in a long follow-up, in some cultural environments, and/or ethnic groups. MHO also has a considerable tendency to convert into MUO, adversely affecting the CVD and overall prognosis. Moreover, obesity, with or without MetS, associates with several other morbidities [29,47]. However, not all MHO subjects become MUO subjects over time, and it would be clinically relevant to determine whether there are some modifiable lifestyle factors that impact the prevalence or duration of MHO subtype.

Our study has some limitations. First, the sample size, in specific in M-O+ group, was small, which may limit the generalization of the results. Thus, long-term follow-up studies with higher cohort size are still needed to confirm the results. Second, due to the study design, many study participants had hypertension. However, diagnosed hypertension was highly prevalent in the Finnish population in the 1990s [48]. Moreover, because AF may be paroxysmal, there may have been non-diagnosed AF cases at baseline and during follow-up. This, however, is a universal limitation in all epidemiological AF studies. The detailed baseline data with echocardiographic measurements and a long follow-up time are the strengths of this study.

In conclusion, according to the present long-term follow-up study of middle-aged Finns, M+O+ and M +O-, but not M-O+, are associated with a greater risk of total mortality and CVD as compared to M-O-, the reference group. Additionally, M+O+, but not M-O+ or M+O-, implies a greater risk of AF. However, M-O + subjects seem to have a considerable tendency to convert to M+O+ over time. Therefore, more long-term studies are needed to establish metabolically healthy obesity as an innocuous condition.

## Supplementary Material

Supplemental Material

## Data Availability

The data used in this study are not publicly available due to health data confidentiality concerns. Possible requests or questions can be sent to prof. Olavi Ukkola.
