# Supplementary material for: Long-term metabolic fate and mortality in obesity without metabolic syndrome
Source: Ann Med. 2022 May 20;54(1):1432–43. doi: 10.1080/07853890.2022.2075915 (PMC9132486; doi:10.1080/07853890.2022.2075915)
Supplement: Supplemental Material [file IANN_A_2075915_SM1399.zip › Supplemental files/Supplementary table 1.pdf]

	M-O+:M+O-	M+O+:M+O-	M+O+:M-O+
**Total mortality hazard ratio**	0.806 (0.522-1.243)	1.126 (0.852-1.487)	1.403 (0.912-2.159)
**Multivariable hazard ratio, model 1**	0.772 (0.499-1.194)	1.115 (0.841-1.480)	1.479 (0.959-2.281)
**Multivariable hazard ratio, model 2**	0.703 (0.432-1.145)	0.952 (0.690-1.315)	1.289 (0.786-2.114)
**Cardiovascular hazard ratio**	**0.420 (0.229-0.770) ****	0.874 (0.635-1.203)	**2.122 (1.151-3.914) ***
**Multivariable hazard ratio, model 1**	**0.404 (0.219-0.745,) ****	0.857 (0.620-1.186)	**2.276 (1.230-4.209) ****
**Multivariable hazard ratio, model 2**	**0.255 (0.125-0.521) *****	0.708 (0.491-1.022)	**2.607 (1.271-5.350) ****
**Atrial fibrillation hazard ratio**	0.803 (0.348-1.850)	**2.072 (1.287-3.335) ****	**2.572 (1.165-5.681) ***
**Multivariable hazard ratio, model 1**	0.655 (0.280-1.536)	**1.970 (1.215-3.195) ****	**2.692 (1.217-5.952) ***
**Multivariable hazard ratio, model 2**	0.585 (0.228-1.497)	1.688 (0.916-3.112)	**2.392 (1.032-5.542) ***

Supplementary Table 1. The hazard ratios of mortality, cardiovascular events and atrial fibrillation between M+O-, M-O+ and M+O+. The hazard ratios are calculated with M+O- as the reference group or M-O+ as the reference group when M+O- is not present. Multivariable hazard ratio model 1 is adjusted for age, sex, smoking (pack years), amount of alcohol consumption and LDL cholesterol. Multivariable hazard ratio 2 is adjusted for multivariable hazard ratio model 1 + hs-CRP, adiponectin and left ventricular mass index (total mortality and cardiovascular events) / left atrial diameter (atrial fibrillation). For event rates, cohort sizes and abbreviations, please see table 2.

* $p<0.05$, ** $p<0.01$, *** $p<0.001$.
