# Supplementary material for: Long-term metabolic fate and mortality in obesity without metabolic syndrome
Source: Ann Med. 2022 May 20;54(1):1432–43. doi: 10.1080/07853890.2022.2075915 (PMC9132486; doi:10.1080/07853890.2022.2075915)
Supplement: Supplemental Material [file IANN_A_2075915_SM1399.zip › Supplemental files/Supplementary table 2.pdf]

	**Original M-O- (n=345)**	**Original M+O- (n=111)**	**Original M-O+ (n=40)**	**Original M+O+ (n=104)**
**M-O- after the control visit (n=194)**	162 (47%)	21 (19%)	4 (10%)	7 (7%)
**M+O- after the control visit (n=181)**	120 (35%)	47 (42%)	2 (5%)	12 (12%)
**M-O+ after the control visit (n=28)**	10 (3%)	6 (5%)	11 (28%)	1 (1%)
**M+O+ after the control visit (n=197)**	53 (15%)	37 (33%)	23 (58%)	84 (81%)

Supplementary Table 2. The persistence of group designation and transfers between groups among the subjects who attended the control visit. Abbreviations: M-O-, no metabolic syndrome or obesity; M+O-, metabolic syndrome without obesity; M-O+, obesity without metabolic syndrome; M+O+, metabolic syndrome and obesity.
